# Autonomous Vehicles Require Socio-Political Acceptance—An Empirical and Philosophical Perspective on the Problem of Moral Decision Making

**DOI:** 10.3389/fnbeh.2018.00031

**Published:** 2018-02-28

**Authors:** Lasse T. Bergmann, Larissa Schlicht, Carmen Meixner, Peter König, Gordon Pipa, Susanne Boshammer, Achim Stephan

**Affiliations:** ^1^Institute of Cognitive Science, Osnabrück University, Osnabrück, Germany; ^2^Institute of Philosophy, Osnabrück University, Osnabrück, Germany

**Keywords:** autonomous vehicles, experimental philosophy, moral cognition, decision making, social acceptance, trolley problems

## Abstract

Autonomous vehicles, though having enormous potential, face a number of challenges. As a computer system interacting with society on a large scale and human beings in particular, they will encounter situations, which require moral assessment. What will count as right behavior in such situations depends on which factors are considered to be both morally justified and socially acceptable. In an empirical study we investigated what factors people recognize as relevant in driving situations. The study put subjects in several “dilemma” situations, which were designed to isolate different and potentially relevant factors. Subjects showed a surprisingly high willingness to sacrifice themselves to save others, took the age of potential victims in a crash into consideration and were willing to swerve onto a sidewalk if this saved more lives. The empirical insights are intended to provide a starting point for a discussion, ultimately yielding societal agreement whereby the empirical insights should be balanced with philosophical considerations.

## 1. Introduction

Autonomous vehicles are currently the most anticipated technological innovation, approaching the capabilities of human drivers and eventually outperforming them. As with any new technology, these developments are faced with controversial issues, in this case particularly with ethical ones. As traffic accidents, due to human failure, are a major source of death and injury in the world, the introduction of fully developed autonomous vehicles (abbreviated as AVs) promises to save lives and reduce suffering. The number of deaths from traffic is staggering, accounting for 2.2% of all deaths or 1.3 million deaths per year worldwide (WHO Website, 2014), making traffic accidents the biggest non-medical source of death and suffering^1^. Each year 4,000 people in Germany die as a result of traffic accidents (Statistisches Bundesamt, 2015) and in the U.S. even 33,000 people (Xu et al., 2016, p.10). Additionally, about 2.3 million people are bodily injured in accidents every year in the U.S. (NHTSA, 2014, p. 1) and nearly 400,000 in Germany (Statistisches Bundesamt, 2016). It is expected that a significant portion of these deaths and injuries could be avoided through the use of AVs. Prima facie, this gives good reasons to further the advancement and introduction of AVs, and provides sufficient motivation to turn toward issues arising in connection with the anticipated introduction of AVs into traffic.

The primary ethical issue dealt with in this text is that of moral decision making: how should an AV behave in situations in which moral reasoning is required? Imagine an AV driving down a street, with parked cars on the one side and a sidewalk on the other. Suddenly a child, hitherto hidden behind the parked cars, steps right in front of the AV. There is almost no time to react, especially not to come to a stop, but the AV could swerve onto the empty sidewalk. It seems clear that from any ethical point of view this is exactly what the AV should do. However, swerving onto the sidewalk simultaneously means to ignore the traffic rule that forbids driving on the sidewalk. Deciding so requires already a non-trivial, but quite common sense, judgment, i.e., that a traffic rule may be nullified in cases where otherwise people would be harmed. To make matters worse, imagine an adult walking on the sidewalk, who would be hit if the AV swerves. This predicament asks for a (tragic) decision whether the life of the child takes precedence over the life of the adult. Alternatively, one might maintain that the pedestrian on the sidewalk is entitled to more protection than a person carelessly stepping onto the street. Clearly, these decisions require ethical assessment, i.e., they need to be made with respect to factors which are recognized by established ethical theories.

In exploring driving strategies in such dilemma situations, we face two opposing skeptical positions with regard to AVs programmed according to ethical standards. First, there are techno-optimists who contest that moral decision making is even a problem. They hold that, since AVs will eliminate traffic accidents and consequently will not get into dilemma situations at all, there is no problem of moral decision making. This view is erroneous. For the foreseeable future accidents cannot be completely avoided and even steep increases in AV capabilities will only reduce and not eradicate the chance of accidents^2^. Although it is not the goal to put AVs in ethically challenging situations, it can not be excluded that they will face such situations. Second, some philosophers question the AVs' capacity for moral agency and conclude that they better not participate in public traffic since AVs cannot be held responsible for what they do in any meaningful sense. The question whether or not AVs are proper moral agents is, however, beyond the scope of this text. As Hevelke and Nida-Rümelin (2015a) suggest, if AVs are introduced, responsibility attributions in traffic have to be rethought. However, even if those attributions are challenged in some way, this does not warrant strong opposition toward AVs. What is important is that they approximate the *behavior* of a proper moral agent and, furthermore, will execute the proper behavior more consistently.

But what behavior of an AV in a dilemma situation would be socially acceptable and, more importantly, what behavior of an AV should be socially accepted since it can be morally justified? Applied ethics is not solely an a priori inquiry. Well reasoned positions need to be developed and intuitions need to adapt to new circumstances. This opens up moral intuitions and social practices as a field of empirical scientific inquiry. More importantly, whether AVs will actually be accepted by society (and their potential to save lives will be realized) may depend on whether they behave in a way acceptable to people. As Bonnefon et al. (2015, p. 8) note: “[…] the field of experimental ethics can give us key insights into the moral and legal standards that people expect […].” In embarking on this experimental investigation, philosophical insights into ethics are not thrown overboard, they inform the experiments conducted, helping to not mindlessly reproduce any human idiosyncrasies. Thus, this paper has the main goal to investigate which criteria are taken to be relevant for the behavior of human drivers in situations which require ethical assessment. In addition it tries to make a first step toward balancing intuitions with socio-political norms and ethical theories. This should provide a starting point for further discussion, with the goal of finding an ethically well informed societal agreement on the issue of moral decision making in AVs.

Skulmowski et al. (2014) show that behavioral studies in a virtual reality (VR) simulation are a suitable tool to investigate people's preferences in *trolley problems*, by replicating the results of questionnaire studies. Data gathered in addition to that published in Sütfeld et al. (2017) provided empirical evidence that people's behavior and the expected behavior in moral dilemma situations are similar in the domain of traffic. The data shows that the decisions made in dilemma situations are strongly correlated with acceptance ratings from observers of these situations. Additionally, this new experimental tool helps to deepen the insight into contextual and situational influences factoring into moral decision making.

## 2. From intuitions to theories and back

The situation described above is very similar to *trolley problems*. The classical trolley dilemma was first introduced as a juridical thought experiment by Welzel (1951) and made popular in philosophy by Philippa Foot (1967, p. 8), who described it as follows: “It may […] be supposed that he is the driver of a runaway [trolley] which he can only steer from one narrow track on to another; five men are working on one track and one man on the other; anyone on the track he enters is bound to be killed.” The trolley rushes toward the five workers, however, the driver could decide to steer the trolley onto the other track. In this case only one worker would die instead of five. How the driver should decide in this situation has been extensively investigated and controversially discussed. Different moral theories come to different conclusions, since they ascribe ethical relevance to different factors. While most theories prescribe steering the trolley to the track on which only one person is working, theories committed to a rigid interpretation of the doctrine of doing and allowing favor inaction. This doctrine will be considered as it is the closest relative of a position sometimes uttered in casual discourse. Some hold the view that an AV should just be programmed to brake and stay its course in these situations. Often people think that this position avoids the problem of moral decision making, failing to recognize that this position itself is an ethical position.

**The doctrine of doing and allowing** includes the commitment to the distinction between intentionally doing harm and allowing harm to happen as a foreseen but unintended side effect of one's action. This position holds that taking action in a situation with only bad outcomes would make one guilty, and therefore the only reasonable course of action is to not act. This implies that it is more justifiable to *let* five people die than to *take action* leading to the death of a single person. Foot's doubts about this doctrine lead to her exploring trolley cases in the first place.

Trolley problems have become very popular in experimental ethics (Christensen and Gomila, 2012; Cushman and Greene, 2012; Waldmann et al., 2012; Greene, 2014b), because they can easily be adapted to focus on different factors to which different theories ascribe relevance. As it turns out most people seem to favor action in Foot's trolley problem (Skulmowski et al., 2014), which means they do not recognize the distinction introduced by the doctrine of doing and allowing in dilemma cases. Other versions of the trolley problem, many of which have been worked out by Judith Jarvis Thomson, trigger different intuitions though. In today's discourse Thomson's version of the original problem is more prominent, where a bystander, and not the driver, can affect the outcome by pushing a lever to redirect the trolley. The most controversial variation is one in which a bystander can affect the outcome by pushing a fat man from a footbridge to stop the trolley (Thomson, 1985, pp. 206–207). The fat-man version places import on whether it is right to use someone to stop the trolley and is considered to elicit intuitions supported by a more current deontological perspective.

**Deontology** does not evaluate the rightness of an action merely in terms of its consequences. It embraces moral norms (e.g., duties), whose conformity to is the right making property. Kant demands that people are not used merely as means to an end. People are considered not as containers for aggregated utility, but in terms of their *humanity*. Rights-based versions of deontology discard Kant's focus on duties. These hold that any violation of rights may not be averted by violating more basic rights. But under certain circumstances it is acceptable to minimize the violation of a specific right. Thomson provides the explication that it is wrong to create a new threat to someone, but it is acceptable to redirect an existing threat to spare lives (Thomson, 1985, p. 1407). This means that using the fat man to stop the trolley is not morally permissible, while it is permissible to pull the lever to steer the trolley onto the other track.

This is often taken to be contrary to a utilitarian approach, which supposedly would allow for the fat man to be pushed as long as it saved more lives. To most people it is less justifiable to use and kill one in order to save five than redirecting a threat to one in order to save five; the duty not to kill seems to override, for most, the positive duty to save a greater number (Greene, 2014b, pp. 113–116).^3^

**Utilitarianism**, the most prominent representative of consequentialism, claims that the moral rightness or wrongness of actions solely depends on the quality of its consequences (depending on the variety of the theory: actual, foreseeable, anticipated or intended), measured by the amount (strength) of rational preferences satisfied. It furthermore commits to the equal consideration of preferences and is focused on the well-being of the moral patients, in the sense that they aim to increase pleasure and reduce pain. Therefore, since the prevention of one death satisfies a smaller amount of rational preferences than the prevention of five deaths, the sacrifice in the trolley problem is seen as morally justified.

The field of experimental ethics (experimental moral psychology) gave rise to a debate about the normative significance of empirical findings. On the one hand it seems reasonable to consider human practices in talking about ethics. Ethical theories that do not recognize factors which seem morally important to people fail to address relevant issues. Therefore, an empirical investigation of people's intuitions and behavior may yield helpful insights into which factors are considered morally relevant (compare Greene et al., 2001; Borg et al., 2006). But on the other hand there is the is-ought-fallacy (also known as the naturalistic fallacy or Hume's fallacy) which motivates much of the reluctance to embrace experimental ethics in general. It seems fairly obvious that *ought* does not follow from *is*. Therefore, one can not infer an ethical theory from people's intuitions, behaviors or brain states measured in an experiment. As the neuro-ethicist Joshua Greene states (Greene, 2010, p. 7):

Like many philosophers, I believe that one cannot derive a substantive moral “ought” from a scientific “is”[…]. More specifically, I agree with Berker that substantive moral conclusions cannot be deduced from scientific findings, neuroscientific or otherwise. Thus, as Berker argues, any valid normative conclusions reached on the basis of scientific research must also invoke one or more non-scientific normative premises. However, it does not follow from this conclusion that scientific results inevitably do “no work” in such normative arguments. […] [S]cientific results can have normative implication—that is, […] they can do important work in normative arguments—without illicitly hopping the is/ought gap.

Greene only uses experimental evidence to cast doubt on the epistemic validity of deontological justifications of moral judgements (Greene, 2015). Accordingly, this paper does not assert that the empirical data accumulated yields normative conclusions. Normative conclusions must be supplied by ethical theories. The empirical investigation only yields which of these theories is more aligned with society's practices and people's intuitions, or more specifically which factors are recognized by people in making moral decision. The empirical investigation may yield certain insights about which theory is preferable, but the normative significance is mainly derived from the theories themselves.

However, such considerations do not elucidate how intuitions and theories play together, since they have been considered to do related but separate work. But there may arise tensions between accounts of guiding principles and judgements in a particular situation. Rawls (2001, p. 18) states:

We can either modify the account of the initial situation or we can revise our existing judgments, for even the judgments we take provisionally as fixed points are liable to revision. By going back and forth, sometimes altering the conditions of the contractual circumstances, at others withdrawing our judgments and conforming them to principle, I assume that eventually we shall find a description of the initial situation that both expresses reasonable conditions and yields principles which match our considered judgments duly pruned and adjusted.

Rawls holds that this process will yield a state in which intuitions (though he avoids this term) and theories are in a balance through a process of mutual adjustment, called *reflective equilibrium*. Through this method not only a single person, but a society can come to a generally acceptable agreement. Such a reflective equilibrium constitutes a moral justification.

The process of reflective equilibrium also yields that a societal agreement may change over time. While a moral decision making procedure for AVs is imminently needed, one shall not assume that an agreement struck will not be subject to future changes. People's intuitions will be shaped by the use of AVs and therefore the equilibrium may shift, which then again has to be adjusted for in concrete implementations.

In the following experiment intuitions about the morally relevant factors in street driving situations were elicited. Thus it covers ethical issues such as the factor of age, whether special protection should be extended to pedestrians on the sidewalk, or whether self-sacrifice is intuitive in extreme situations. The elicited intuitions can be compared to related social or political norms. A case in point is the recent report of the German ethics-commission concerned with autonomous driving (BMVI, 2017), the task of which was (amongst other things) to formulate norms for the implementation of decision procedures in autonomous vehicles. Since the experimental data were collected in Germany, we have a good measure whether the norms specified are coherent with subjects' intuitions. It presents a challenge to these norms, if they are not representative of a state of reflective equilibrium. In the following experimental evidence will be presented and contrasted with a number of potential guiding norms, to see whether these norms can be justified by a reflective equilibrium.

## 3. Methods

The VR experiment we conducted put test subjects in the position of making choices between two lanes on which their vehicle drove at a constant speed. The test subjects drove along different roads until obstacles emerged on both lanes, upon which they had four seconds to switch the lane they were driving on (or not), finally hitting a person. To conform to ethical guidelines in experimental studies, the VR simulation did not show the actual accident, but went black shortly before the collision. Since the collision was merely implied the subjects could not be certain whether the persons standing on the road were injured or killed. Therefore, terms like “killed,” “injured,” or “hurt” will be used loosely throughout the paper. To avoid biases based on gender or other factors^4^, the person were all very similar looking men (unless otherwise indicated). The experiment featured a number of ethically challenging situations, which each subject had to go through in a randomized order (also the lane on which the obstacles were presented and the starting lane of the vehicle were randomized). All participants (in the final sample) completed all of the different trials and none of the trials were presented more than once. The discussion of the situations will be divided into groups which each illuminate a different aspect of problematic social interactions in the context of autonomous driving. The situations are designed such that the options the subjects face correspond to opposing positions, one may adopt toward traffic dilemmata.

The study tested 216 subjects, of whom 27 were excluded (15 aborted the experiment, 12 underperformed in training trials); of the remaining 189 subjects, 62 were female and 127 male. The age of the participants varied between 18 and 67 (24.32 years on average). Participants were tested in multiple locations to acquire a more diverse sample. One-hundred-forty-two were tested at Osnabrück University, either recruited through standard student participant acquisition, approached on campus or recruited at a student information event (these students received experimental hours for their respective degrees if applicable and desired). The other subjects (74) were recruited and tested at the city hall and a local car inspection authority. All subjects were tested with the same equipment. Active noise canceling headphones were used to exclude differing ambient noise as a distractor.

Experimenter-subjects interaction was kept to a minimum. Before the experiment started, experimenters checked whether subjects had previous trauma relating to traffic, mental disorders or visual impairments. Subjects were also advised how to abort the experiment and informed that they will face problematic driving situations (or experience nausea due to the VR-device). Furthermore, subjects were (truthfully) informed that experimenters were unable to monitor any decision made inside the VR environment. Experimenters assisted subjects in putting on the equipment, i.e., made sure VR-device and headphones were put on correctly, as well as making them familiar with the basic functionality of control elements.

All experimental instructions were given within the VR simulation. Subjects were instructed that they were the only passenger in the vehicle. They were also informed of their limited control over the vehicle, i.e., that they could not change speed or divert the car from the two lanes intended for driving. Subjects could only steer from one lane to another, and the vehicle always aligned itself to the lane steered to. Additionally, they were instructed that audio cues indicated that control over the vehicle was handed over to them or that a rising sound before a collision indicated that control was being revoked. Subjects were not instructed to behave in any particular manner. To make subjects familiar with the controls of the vehicle in the VR environment, subjects had to avoid pylons in training trials, including control transition cycles. They had to successfully avoid three pylons on different lanes; if they were unsuccessful, the training trial repeated. Three training trials were conducted in the three different environments utilized in the subsequent experimental trial. After successfully completing the training, the subjects were introduced to the different kinds of obstacles they would face in the following trials (man, elderly man, child, kneeling man, chasm). Then subjects could continue to the testing phase where they were presented with all trials reported on below in a randomized order. After completing all the trials biographical information was collected inside the VR environment. Subjects took about 14 min on average to complete the experiment inside the VR environment.

All trials were kept as uniform as possible. Three road environments were implemented in the unity engine to allow for a plausible environment for the different experimental conditions: a suburb with vehicles on both sides to make driving off the road to avoid obstacles impossible, a city with one lane constrained by vehicles on one side and a sidewalk that could be driven onto constrained by buildings and a mountain environment constrained by a rock face on one side and a railing (protecting from a steep drop) on the other. The salience of the sidewalk in the city environment was increased by a sound and tilting of the vehicle when it drove over the curb (furthermore the driving sound when on the sidewalk was slightly different indicating that subjects were not driving on the normal road). The vehicle drove with a constant speed of 36 kph (10 m/s), while a fog-like curtain restricted the view of the subjects to 55 m ahead. Subjects drove along a short stretch of road at some point encountering the critical situation. Each trial began driving 160–200 m before the obstacles on the road (distance was kept inconsistent to avoid subjects expecting the precise moment where they first saw the critical situation). This gave subjects 4 s (40 m) to make a decision before driving control was revoked from them 15 m before the potential collision. Control was revoked at this point to preclude subjects from attempting to steer in between obstacles through incomplete lane changing maneuvers. Five meters before the collision the screen went black, to avoid a visual display of the collision (precluded by ethics guidelines). The screen remained black for two seconds until the next trial began.

### 3.1. Dataset 1: the classic trolley problem

In our experiment we tested whether subjects would make an active choice to effect minimal loss of life, i.e., whether they recognize the number of people affected as a relevant factor. Participants driving the car in the VR-simulation had to decide in three trials between driving on a lane with one person or another lane with 2, 4, or 6 people, respectively. We only presented standing adults as obstacles. We hypothesized that people would act in favor of saving more people. Due to the structural similarity with the switch version of the trolley dilemma, it was expected that people's behavior would be in line with common intuitions, that acting to save more people is the right thing to do in this case, rather than remaining inactive.

#### 3.1.1. Results and discussion

**Figure 1:**
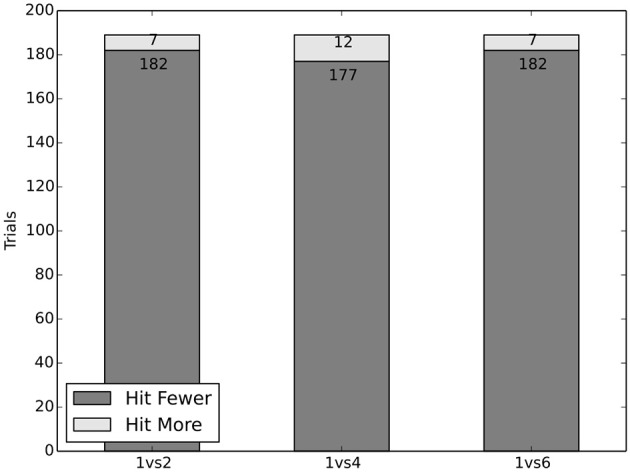
Results of classic trolley dilemma were replicated.

The empirical data supported our expectation: The experiment's subjects acted in these trials highly in favor of action (or inaction, in case they were already on the lane with only one person). In 95.4% of the trials participants chose to drive on the lane on which their vehicle would hit the single person (see Figure 1). This behavior was independent of the starting lane of the vehicle and the side of the road the people were standing on. It was only sensitive to the different group-sizes in terms of effecting minimal loss of life. The specific group-size does not seem to be a factor.

One may infer that these results support the claim that people do not conform to the rigid interpretation of the doctrine of doing and allowing used here, which would favor inaction. This is consistent with both consequentialism and deontology. In the given situation it is mandatory from the perspective of both positions to try to save as many lives as possible, as there is no way to avert harm in any case, and therefore they are congruent in their desired behavior. The report of the ethics-commission cautiously formulates a similar norm (BMVI, 2017, p. 11, pp. 18–19): “A general implementation aimed at the minimization of bodily harm could be justified. (Translation by the authors.)” Since moral theories are in agreement and intuitions are on their side, this provides an important first step for the decision making of an AV: A consideration of the number of lives is justified. The AV should effect minimal loss of life, if this loss can not be avoided altogether and no new threat is introduced to do so.

### 3.2. Dataset 2: egoism vs. altruism

The last section discussed the classic and well covered (both experimentally and theoretically) version of the trolley problem. But what if a situation arises in which the only possible way to save a group of people is to risk one's own life? Thomson also realized the absence of a self-sacrifice choice in the classical dilemma and suggested a 3-track version of it by adding the option to let the trolley drive over the person who pulls the switch instead of the five people or the one person (Thomson, 2008).^5^ In the situation, in which an AV has to either kill several people in front of it or the person inside of the AV, classic utilitarianism mandates sacrificing oneself. This demand however does not fit those people's intuitions who would prioritize self-preservation, and who may reasonably hold that this utilitarian demand is excessive. Some may even hold that it is morally right to opt for self-preservation, which may be supported by moral egoism. Although it is debated whether this is an ethical theory proper.

On the deontological side the demands aren't as clearcut. Thomson herself is not sure what exactly deontology would prescribe and ultimately leaves the question open (Thomson, 2008, p. 371). Nevertheless, if we consider human behavior to get a guideline for how an AV should act, it is highly informative to look at situations involving self-sacrifice, because they bear the potential for much stronger disagreement between people than those considered in dataset 1: “The Classic Trolley Problem.”

In our experiment we investigated dilemma situations with the option for self-sacrifice in a mountain road-like VR environment: The subjects drove along a road, bounded by a rock face on the one side, and a steep drop on the other. Suddenly a group of people appeared and they had to chose between driving against them or driving toward a large chasm, risking their own life. The group size varied between two and seven; each subject faced each group size.

#### 3.2.1. Results and discussion

**Figure 2:**
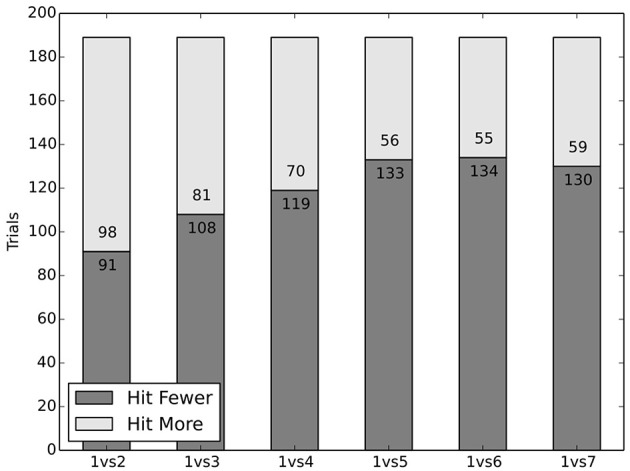
Self-sacrifice is surprisingly common.

The participants acted more altruistically than expected, though the results depend on the number of potential victims in a trial. Even two persons were saved in more than half of the trials. As the number of people rises, the number of subjects choosing to save them rises, too. While two people were saved in 52% of cases, three people were saved in 57% of cases and four people were saved in 63% of cases. In equal measure, groups of five, six and seven people were avoided in roughly 70% of cases (see Figure 2).

The results lead to the conclusion that there are different groups of decision makers, committed to different strategies^6^: One group consistently chose to hit other people to save themselves and may therefore be labeled (moral) *egoists*. A second group always chose the option of self-sacrifice, therefore labeled *altruists*. A third group, labeled *switchers*, changed their decision behavior depending on the group size, opting for self-preservation in trials with fewer people and self-sacrifice in trials with more.

The driving behavior of the *egoists* indicates putting more weight on self-interest. They seem to value their own lives, and the consequences this loss would have, more than any (tested) amount of other persons' lives. Comparable attitudes can be observed on the streets: Among other things, people who buy an SUV often do so to protect themselves even at the expense of the higher risk of injuries to other parties. The number of egoists, however, was unexpectedly small, encompassing only 20% of subjects.

The second group showed altruistic behaviors. In this context this fits utilitarianism, since in all trials in which self-sacrifice was an option, the number of people saved by sacrificing oneself numerically outweighed the test subject driving the car. Altruists were (surprisingly) the most common group of decision makers, accounting for 39% of subjects.

The third group of subjects, the *switchers*, changed their decision behavior depending on group size. They sacrificed themselves only if the group size reached a certain threshold; otherwise they opted for self-preservation. The behavior of this group can not easily be mapped onto any particular moral attitude. It may be explainable though in terms of conflicting cognitive processes, one concerned with saving one's own life, the other with more utilitarian considerations—the second process taking over if the threshold is reached. Alternatively, it may be a single consequentialist weighing process in which the subject takes herself more into consideration than others, valuing her own life more by a certain factor. Switchers accounted for 17% of participants: 4.8% of all subjects switched between two and three people, 5.3% switched between three and four people, while 3.7% switched between four and five people.

The missing 24% of subjects could not definitively be associated with one of the groups, as their behavior was not perfectly consistent with the behavior defining the groups; a subject for example who always behaved altruistically except in one case, say, with three or four persons, was not assigned to one of the three groups.

The results of this condition are controversial. Since in all cases the majority of subjects chose self-sacrifice and the altruist group was the largest group of decision makers, encompassing more subjects than the other two groups combined, it seems that most subjects do not recognize themselves as a factor that deserves additional moral attention. In this respect utilitarianism is consistent with the behavior exhibited by most participants. But a large minority of subjects did save themselves. Deontology seems to allow for both, saving oneself and saving others, and therefore is consistent with both behaviors. It seems, however, that a decision-procedure should yield a definitive course of action.

Ethical theories, however, are not necessarily concerned with providing a definite course of action. As it happens utilitarianism does yield a definite course of actions, but most other theories provide a framework, defining what is allowed or forbidden—leaving it up to the agent to find the right action within such a framework. Nevertheless it seems highly suspect to leave it up to the computer to find the right action within a moral framework—a computer needs to be taught how to find the right action. This makes comparing ethical theories somewhat less appealing, as the need for a definite procedure favors utilitarianism not on ethical, but on technical grounds. Thus, theories may provide the motivation for looking at a specific factor, as a potential disagreement in theories suggests that it may be interesting. However, this does not necessarily endorse the theories committed to this factor.

The German ethics-commission is fairly adamant on this question, stating that it would be incompatible with the human dignity to expect people to sacrifice themselves in unavoidable dilemma situations (BMVI, 2017, p. 19). Thus an AV should not be programmed to sacrifice its passengers.

A societal agreement may require a compromise, e.g., that AVs protect their passengers more than other people, but will save others if this will clearly save more lives. It seems that people show a remarkable cognitive dissonance about this issue, wanting others to use AVs that would sacrifice their passengers, but themselves prefer to use AVs that would safe them (Bonnefon et al., 2016). This is a classic case of short-sighted self-interest vs. the common good. Car manufacturers need to tailor their product to the consumer. Since the consumers would be the ones sacrificed, they may not conform to general societal considerations. This in turn may prompt manufacturers to use protection of passengers as a selling point (or even worse sell more self-preserving software to wealthier clients). Like in many prisoner's dilemma like situations, every person may prefer AVs which protect herself first, but overall it would be better for everybody if AVs protected the interests of all. This emphasizes the importance of a societal agreement for a uniform decision procedure, to prevent consumers from facing a prisoner's dilemma situation. It may however be prudent to study situations involving self-sacrifice more intensively, which may provide insight into the psychological tendencies involved, to find the most acceptable solution. Generally speaking it is clear that intuitions, norms, and theories are incoherent in the case of self-sacrifice and thus claims about such cases lack justification.

### 3.3. Egalitarian troubles

While the previous dataset problematizes the relationship between individuals' and societal concerns, the following dataset is concerned with problems of equality between different groups. Two opposing positions are often invoked—egalitarian treatment and egalitarian consideration. Egalitarian treatment demands that people are not treated differently according to certain characteristics (such as age, skin-color, disability, gender, or wealth), whereas egalitarian consideration demands that treatment is balanced by considering everyone's well-being equally.

Although many studies show that moral behavior is often based on utilitarianism (Greene, 2007), this (arguably) does not hold for all distribution scenarios. Baron (1994), for example, investigated whether people would support cutting back on a balanced healthcare system in virtue of better caring for a large sub-group, if this would lead to a better healthcare in general. Most people would not agree with such a decision. This is supported by the findings of Dawes et al. (2007), who found similar preferences concerning income distribution. In both cases people preferred a more equal distribution instead of maximizing the overall welfare through unequal distribution.

#### 3.3.1. Dataset 3: the influence of age

Problems of distribution also arise in the context of AVs. A societal agreement may be hindered by the widespread disagreement about how to exactly spell out egalitarianism. In the third experimental condition we tested how people decide if they had no other option than to hit either children, elderly persons, or adults. As before, the subjects drove along a two-lane road, eventually encountering two different types of people standing on the road, one on each lane. The pertinent question in these trials is whether age, i.e., the remaining lifespan of the people involved, is a relevant factor in such decisions. Egalitarian treatment would not allow for such a factor to be considered, as it would be a form of age-based discrimination, while egalitarian consideration would allow for this factor to be relevant, as in a longer lifespan more well-being can be enjoyed.^7^

#### 3.3.2. Dataset 3: results

**Figure 3:**
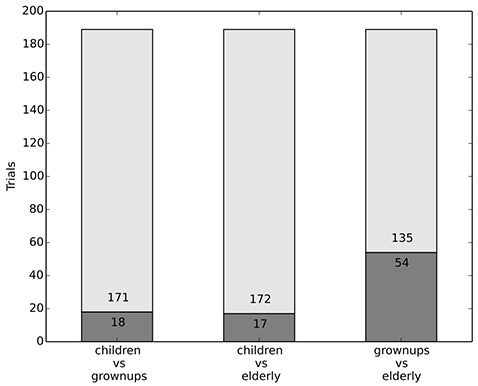
Age of people is an important factor.

The results of our experiment are clear: Children were saved a lot more than all other persons and adults more than elderly persons. The saving rate of adults and elderly persons do not differ substantially when they were paired against a child. In both cases about 90% of the participants chose to save the child and hit the adult/elderly person. Furthermore most of the participants (72%) chose to save the adult and hit the elderly person (see Figure 3).

In all these scenarios the decision behavior is clearly matching the view of equal consideration rather than equal treatment, which would not allow to take the age of people into consideration. The results indicate that people are more prone to save younger people, which is in accordance with a (more general, questionnaire based) study of Kawai et al. (2014). Our results suggest that strict equal treatment is not in line with people's intuitions, especially if it concerns children.

#### 3.3.3. Dataset 4: the influence of likelihood of injury

In another trial we investigated whether the likelihood of injury is a relevant factor in the participants considerations. Thus, in addition, subjects were faced with a kneeling adult. The underlying hypothesis is that the lower position of the man's head, would make him more susceptible to injury.

#### 3.3.4. Results

**Figure 4:**
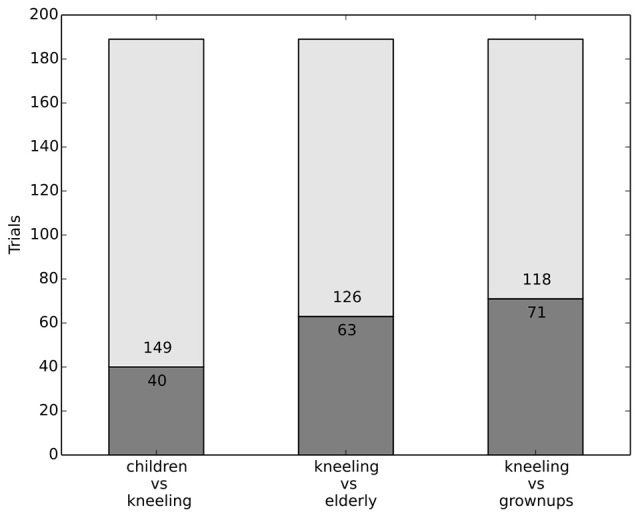
Relative risk of injury seems to play a role.

The kneeling person was saved more often in the conditions with adult and elderly persons (62% saved him in place of an adult, 67% in place of an elderly person), which suggests that he is entitled to more protection (see Figure 4). This better outcome for kneeling people extends also to the scenario with the child and kneeling adult: the child was saved less often (79% of the participants), as compared to the condition with a child and a standing adult (90%). So the decision behavior can again be seen as a non-egalitarian treatment, as people are treated not equally, but with deference to the likelihood of injuries.

#### 3.3.5. Discussion dataset 3 and 4

The results in this section indicate that subjects' behavior is sensitive to age of the obstacle and (to a lesser degree) the likelihood of injury. Since subjects show some deference to likelihood considerations, it should be investigated how exactly likelihood considerations factor into a decision procedure. Further research on likelihood considerations should also more generally investigate how risk (as a likelihood of accident judgement) is balanced with the desire to reach a destination more quickly. Age, however, is an often considered factor. It seems that in a more general societal context people would be less comfortable with distinctions based on age. Thus, positions need to be balanced such that they not only capture the behavior in dilemma situations, but also correspond to people's preferences for a just society, in general. The ethics-commission took a clear stance on this issue, prohibiting various forms of discrimination. They considered taking the age of victims into consideration to be a form of discrimination (BMVI, 2017, p. 11). This is especially noteworthy as according to the German constitution age-based discrimination is not recognized as illegal discriminatory practice. It is clear, however, that norms and intuitions diverge on the issue of age and thus there is no equilibrium.

### 3.4. Dataset 5: sidewalks and innocent bystanders

This paper started with the introduction of an example, featuring the possibility of saving a child by swerving onto a sidewalk, hitting another person. While the last section investigated whether special protection would be extended to children, the relevance of the sidewalk is still an open question. In general, people on a sidewalk have a reasonable expectation of safety, which is enforced by traffic laws, i.e., they have the right to additional protection. Traffic is regulated trough a plethora of rules and regulations, furthering safety, but also ensuring that all participants in traffic can reasonably expect certain behaviors.

To test whether in fact people recognize specific rules, specifically the prohibition of driving on a sidewalk, as morally relevant factors, we confronted the subjects in six trials with the situation of driving on a one-lane street toward a group of adults (varying in size between two and seven), who could only be saved by driving on the sidewalk instead, hitting a single person there.

#### 3.4.1. Results and discussion

**Figure 5:**
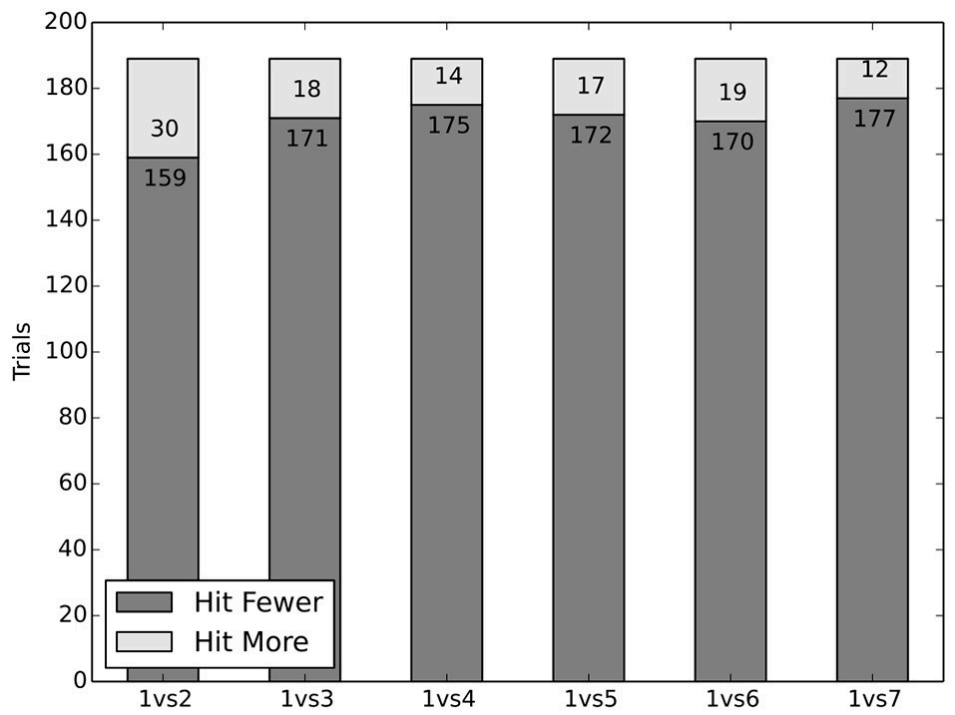
Subjects opted to hit fewer people, not to avoid sidewalk.

As the data reveal, the sidewalk had little effect in comparison to the initial trials without a sidewalk (compare dataset 1: “The Classic Trolley Problem”), where subjects safeguarded the greater number of lives. In roughly 90% of trials, subjects ignored traffic rules when having the chance to save a greater number of people. Whilst for a group size of two people, 85% swerved to the sidewalk; for a group size of three and greater this was done in about 92% of trials (see Figure 5). Overall only 2.6% of subjects never drove on the sidewalk.

The data in this section suggest that people do not recognize the moral relevance of the sidewalk being a safe place for pedestrians when it comes to dilemma situations. Nevertheless, their behavior seems to be justifiable from both a utilitarian position, as this choice preserves more well-being, and from a deontological position, since swerving onto a sidewalk minimizes harm (as long as pedestrian on the sidewalk are not considered innocent bystanders).

However, implementing such a decision procedure for an AV could lead to some dodgy situations. Hevelke and Nida-Rümelin (2015b) for example raise the issue of two people carelessly walking on a street, right in front of an AV. The AV could now swerve to the sidewalk hitting another person, but should it? Intuitively it seems unfair that the pedestrian on the sidewalk should have to suffer because of the others' inattentiveness. Further studies should show how people behave in situations which make it more explicit that the people on the street were inattentive, reckless, or malicious.

The ethics-commission formulated a clear stance on this issue, that those who create traffic risks are not to harm those, who do not create traffic risks, in any way. This holds even if this would save more lives or would save the passengers of the vehicle (BMVI, 2017, p. 10). This is a clear prohibition on sacrificing people on the sidewalk, since they can not reasonably be construed to create a traffic risk.

The experimental evidence, again, points to an incoherence between norms and intuitions. Though prudentially the norm not to sacrifice people on the sidewalk seems well justified, a lack of experimental support raises some questions.

### 3.5. Experimental limitations and further research

Even though VR simulations have a higher ecological validity (as opposed to for example questionnaire studies) the moral significance of real life situations is probably not fully captured in such a simulation. Especially self-sacrifice conditions may be confounded due to a lack of actual survival responses (missing survival responses, however, may allow for moral rather than instinctual responses). Additionally, certain decision making responses may be cognitively tied to the perception of factors not included in the simulation, e.g., specific intentions could not be ascribed to the static person models (on relevance of intentions see Borg et al., 2006; Greene et al., 2009).

Another limitation may be that subjects infer information from the simulated environment. However, one can not be sure that they did not infer the wrong information. For example, many trials were conducive to simple mathematical reasoning, which may have suggested to subjects that this is a desired kind on reasoning in this experiment. Order effects in trolley problems have been reported on by Wiegmann et al. (2012) and Schwitzgebel and Cushman (2012). However, the effect was only present when “non-intuitive judgements” preceded “intuitive judgements.” Thus, the prevalence of mathematical reasoning in this study (which is a kind of “intuitive judgement”) should not have interfered with possible “unintuitive judgements.”

Although a third of the data was collected from “people on the street,” the population sample of the study may still be not representative enough. The gender and age bias was mainly due to recruitment constraints. Students especially were easier to recruit and schedule experiments with, resulting in a strong overrepresentation of a younger age-group. These biases, however, do not undermine the validity of the claims made.

Further studies will elucidate the question of perspective effects on moral evaluations. In this study subjects were always in the position of the driver, in an ongoing study it will be investigated whether the concrete perspective a subject is put into (passenger, pedestrian, onlooker) plays a role in their moral evaluations.

## 4. Conclusion

Throughout this paper empirical insight into human driving behavior in morally problematic situations was discussed. The main goal is to find out which factors are recognized as morally justifiable. The motivation for this being that the pending adoption of AVs into traffic will lead to situations in which these AVs will have to make decisions which previously were only made by human moral agents. Since moral insight does not come naturally to an AV, decision criteria and procedures need to be programmed into every AV. These programs, however, need to be socially acceptable and morally justifiable. While empirical investigation helps to fathom what is acceptable to people and society, the ethical permissibility of such criteria require a mutual adjustment between (what has been called here) intuitions, political norms and ethical positions, with the goal of finding an equilibrium, i.e., well reasoned criteria. This process is just in the beginning and this paper aims to provide a first step in such a discussion and direct future research toward areas, that show strong tensions between different positions. The empirical data shows that political and social norms are for the most part not yet coherent with intuitions. These areas will need further study and public discussion to allow for mutual adjustment, which would bring about a strong justification based on a reflective equilibrium.

In dataset 1: “The Classic Trolley Problem” a basic variant of the trolley problem was explored. Subjects were forced to choose between hitting a single person or a group of people. The overwhelming majority (~95%) decided to hit the single person, clearly showing that the number of potential victims is a relevant factor in peoples' decisions. This is supported by utilitarian and deontological theories, as well as political norms, and therefore this insight may provide a cornerstone for a moral decision making procedure. Such norms, however, usually are moderated by certain restrictions, specifying which people should be considered in these kinds of situations or which factors are allowed to be considered. The following datasets will investigate intuitions about these demarcations.

Dataset 2: “Egoism vs. Altruism” explored the issue, whether subjects would rather sacrifice themselves to let other people survive or preserve their own life at the expense of others. Utilitarian theories do not recognize the agent's preferences as a more important factor than other persons' preferences. This means that in a situation where more people are harmed by a self-preserving action, utilitarianism demands self-sacrifice. This is a rather harsh demand, and may be open to the demandingness objection. Nevertheless, roughly 70% of subjects chose to sacrifice themselves rather than drive over five, six or seven people, while fewer subjects sacrificed themselves for four (63%), three (57%), and two (52%) people. The subjects seem to break down into three groups of decision makers; the *egoists* (20%), *altruists* (39%) and *switchers* (17%). Egoists never chose to self sacrifice, while altruists always self sacrificed. Nevertheless, the majority of people seem to not take themselves to have a special moral status. This may also be cautiously thought of as an endorsement of utilitarianism which places equal relevance on the agent and others affected, while deontology does allow for both behaviors. However, in the actual implementation of an AV a definite behavior needs to be programmed. The political perspective, as laid out by the ethics commission, is to forbid AVs from sacrificing their passengers. Their position is understandable from a legal and commercial aspect, but is not strongly reflected in the intuitions of people.

Datasets 3 and 4 examined whether subjects considered age and likelihood of death (or injury) to be relevant factors. The experiment confronted subjects with the decision between two people, one on each lane, either a child and an adult, a child and an elderly person, or an adult and an elderly person. While equal consideration views are sensitive to the age—i.e., the future lifespan—of the people killed, because death takes away future pleasures or violates preferences about the future, equal-treatment-views usually don't allow for people to be treated differently depending on age. The empirical evidence shows that subjects take age into consideration as a relevant factor; subjects protected children over adults (90%) and elderly (91%), and furthermore protected adults over elderly (72%). However, this question gives rise to substantial disagreement and taking age into consideration may be a form of agism—which was explicitly forbidden by the ethics commission. Subjects were furthermore presented with a kneeling person on one lane and on the other either a child, an adult, or an elderly person. The assumption is that the lower positions of the kneeling persons head would make them more susceptible to death and injury. Subjects seemed somewhat sensitive to this factor, suggesting that it may be relevant to some degree.

Finally, dataset 5: “Sidewalks and Innocent Bystanders” investigated whether subjects adhere to the rule not to drive on a sidewalk. Swerving onto the sidewalk to save more people may seem justified for that reason, but conversely there are good reasons to recognize the special expectation of safety pedestrians on the sidewalk have—they can reasonably be considered innocent bystanders. Subjects were confronted with the choice of driving over a group of people (between two and seven people) or hitting a single person on the sidewalk. Even though driving on a sidewalk is explicitly forbidden, roughly 90% of subjects chose to ignore the traffic rules—implying that the sidewalk itself is not a relevant factor. The protection of innocent bystanders and thus people on the sidewalk, however, is one of the cornerstones of the ethics commission's rules. This shows another critical tension.

Throughout the experiment the subjects' decision making is more in line with utilitarianism, i.e., utilitarianism is more sensitive to the factors which subjects seem to recognize as morally relevant. However, consequentialist theories are often taken to be moderated by deontological theories, so it is important to point out that the decisions made by subjects (for the most part) are at least allowed by deontological theories. This should, however, not be taken as an endorsement of any ethical theory. Utilitarianism is more in line with experimental evidence, because it provides a definite course of action, but this is not necessarily the goal of ethical theories. Ethical theories merely provide the motivation for investigating certain, potentially relevant, situational factors. The factors which are featured in human decision making are good candidates for factors that should also be featured in a decision procedure for AVs to maximize their acceptance. The factors identified here, however, clash not only with moral theories, but also with political norms as laid out by the German ethics commission for autonomous driving. The contrast between data and norms, show which positions in the debate are on solid footing and those which lack a coherent justification.

The tentative cornerstone of such a decision making procedure is the consideration of the number of victims, aiming to minimize them. While the agreement on this norm is solid, the question arises, whether all possible victims that could be included in this calculation should be included. Three areas were considered and show a strong disagreement between political norms and intuitions: the question of self-sacrifice, the consideration of age, and whether pedestrians on the sidewalk should be granted special protection. The divergence of intuitions from norms shows that the societal debate on this issue is only just beginning. The reasonableness of political norms needs to be communicated and discussed in society. If this does not yield significant changes in the moral attitudes people display, the norms need to be revisited. It seems imperative, however, that the norms guiding the decision making of AVs need the strongest possible justification, thus neither going with a decision procedure based solely on intuitions nor implementing one based solely on political concerns should be considered a good solution to the problem.

## Ethics statement

The study was approved by the ethics board of Osnabrück University. We adhered to APA and national guidelines. We did not test any minors and informed all participants that they should not participate, if they had neurological or psychological problems. Participants were also informed that they shouldn't participate if they experienced any trauma in a driving situation. (Also participants with visual problems were excluded for experimental reasons). Participants were informed of this at the point of recruitment, verbally by the experimenter and had to sign these guidelines on their consent form. They were additionally informed that they were free to abort the experiment at any point, if they experienced any nausea from the VR device or felt uncomfortable in the situations presented to them.

## Author contributions

LB, LS, CM, PK, GP, and AS were involved in the design, setup, and analysis of the study. LB, LS, and CM wrote an early version of the paper. LB and AS were in charge of subsequent revisions. SB contributed substantially on final revisions clarifying the role of ethical theories.

### Conflict of interest statement

The authors declare that the research was conducted in the absence of any commercial or financial relationships that could be construed as a potential conflict of interest.

## Footnotes

^1^Numbers of that magnitude tend to be hard to comprehend and 2.2% of deaths may seem like a negligible amount. If one considers, though, that the annual number of, e.g., homicides is about 437000 worldwide (United Nations, 2013, p. 13), and that thus the reduction of even just one third of the overall amount of traffic deaths would equal an end to all murder, it becomes clear that the reduction of 2.2% of deaths is not negligible at all.

^2^This point has been elaborately argued by Goodall (2014). Furthermore, there were two noteworthy incidents in the last year: The minor accident of a Google driverless car, where it misjudged the behavior of a bus driver and a minor collision ensued (Google, 2016). And the tragic death of a Tesla driver, who put his Tesla in “autopilot” mode (which is still an experimental feature, usable at one's own risk). The Tesla autopilot failed to recognize a truck and collided with it (Tesla, 2016).

^3^One may also interpret this finding in such a way that people employ consequentialist and deontological reasoning depending on the degree of personal involvement, i.e., personal force and/or spatial distance. This observation made trolley problems useful for psychologists engaged in the empirical study of moral cognition. Notably, neuroscientist and philosopher Joshua Greene investigates moral reasoning in the context of trolley dilemmas using fMRI-experiments. According to Greene's dual-process theory of moral judgment, moral reasoning is influenced by both automatic emotional responses and by a controlled, conscious reasoning manual mode. Greene assumes that “characteristically deontological judgments (i.e., generated by a prioritization of people's rights and duties) are preferentially supported by automatic emotional responses, while characteristically consequentialist judgments (i.e., derived by impartial cost-benefit reasoning) are preferentially supported by conscious reasoning and allied processes of cognitive control. Characteristically deontological judgments tend to be driven by emotion and typical consequentialist judgments by cognitive processes” (Greene, 2014a, p. 717). Greene assumes that the tension in ethics between deontology and consequentialism is a manifestation of the tension in cognitive design between efficiency and flexibility. Automatic settings, such that according to Greene trigger deontological judgements, could therefore function well only when they had been shaped by (evolutionary, cultural, or personal) trial-and-error experience (Greene, 2014a, p. 714). And indeed, Greene and his colleagues could show in neuroscientific experiments that the contemplation of personal moral dilemmas like the one in which one needs to push a bystander produce stronger neural activity in brain regions associated with emotional response than the contemplation of impersonal moral dilemmas like the one in which one needs to steer the trolley (or in our case a car). The contemplation of impersonal moral dilemmas is correlated with a stronger activity in brain regions associated with higher cognition (Greene, 2007, p. 43–44).

^4^Other studies, not utilizing a virtual reality environment, also introduced the significance of the relationship between the agent and the people to be killed/saved by letting the participants imagine the people on the tracks were close relatives or members of the same (cultural) group (Petrinovich et al., 1993; Lucas and Livingston, 2014; Swann Jr et al., 2014).

^5^Even though Thomson only intended to use this hypothetical trilemma situation to get new insights into the classical 2-track version of the trolley problem, which Di Nucci (2013) provided experimental support for, Huebner and Hauser (2011) used Thomson's design to test laymen's intuitions about self-sacrifice. But for the majority of participants (81.7%) letting five people die was not an option, judging this dilemma to be about whether or not they would sacrifice themselves for only one person. This is an interesting result but does not tackle the question of how people would decide in a forced choice situation.

^6^Since human subjects usually make some mistakes in psychophysical experiments, not all subjects neatly conformed to these groups. To support the assertion that there are three groups of decision makers, we tested how many data points were inconsistent with this hypothesis. The result was that about 5% of the data-points were not explained by this hypothesis, which is in line with the expected error rate in psychophysical experiments. This suggests that the underlying assumption about three decision strategies is justified.

^7^A way of calculating these future pleasures on an average is to quantify it with the help of quality adjusted life years (QALYs). This measurement is also used in healthcare decisions to gather the amount of “full quality years” a person will be able to spend according to statistics and condition of the person. If a child is hit and killed, more QALYs are lost than if it is the adult who dies, because a child on average has more life ahead of it. An utilitarian behavior which measures utility based on the QALY would consider this and thus rather protect the younger.
